# Financial strain among West-Javanese parents: its association with marital satisfaction and quality of life, and the role of dyadic coping

**DOI:** 10.3389/fpsyg.2024.1434426

**Published:** 2024-09-16

**Authors:** Langgersari Elsari Novianti, Fredrick Dermawan Purba, Johan C. Karremans, Hendriati Agustiani

**Affiliations:** ^1^Faculty of Psychology, Universitas Padjadjaran, Sumedang, Indonesia; ^2^Center for Relationship, Family Life, and Parenting Studies, Faculty of Psychology, Universitas Padjadjaran, Sumedang, Indonesia; ^3^Behavioural Science Institute, Radboud University, Nijmegen, Netherlands

**Keywords:** dyadic coping, financial strain, marital satisfaction, quality of life, family with school children

## Abstract

In the past decade, the concept of dyadic coping as a buffer against stress in romantic relationships has received much attention in Western countries, but it has rarely been studied in non-Western countries and among parents with school-aged children. The aim of the present study was to investigate the moderating effect of dyadic coping on the link between financial strain and marital satisfaction, as well as the mediating effect of marital satisfaction on the relationship between financial strain and quality of life. There were 751 heterosexual couples whose eldest child aged 7–12 years in West Java, Indonesia (mean age husband = 37.53 SD = 5.09; mean age wife = 34.42 SD = 4.85) fulfilled the paper and pencil questionnaires in the study. The moderated mediated model illustrated that (1) greater levels of dyadic coping weakened the negative association between financial strain and marital satisfaction for husbands and for wives (2) for both husbands and wives, there were no mediation effect (3) for both husbands and wives, financial strain was negatively associated with quality of life; and marital satisfaction was positively associated with quality of life. We discuss both the theoretical and practical implications of these findings.

## 1 Introduction

Families with school-aged children experience a myriad of challenges that can be stressful and affect both partners, their relationship, and their children (e.g., Douthitt and Fendyk, 1988; Browning, 1992; Webley et al., 2001; Pollak, 2002; McGoldrick et al., 2016). One particular factor that can cause stress and have a detrimental impact on parents is financial strain, defined as the state of distress, anxiety and worry, or feelings of not coping, created by economic or financial events; the term financial strain is synonymous with financial/economic hardship, financial/economic stress, or financial difficulties or inability to cope financially (French and Vigne, 2019). Financial strain occurs when households perceive their financial resources as insufficient to meet their financial obligations and needs. Several previous studies have demonstrated that financial strain can negatively affect the well-being of the relationship as well as the well-being of both partners (e.g., Vinokur et al., 1996; Williamson et al., 2013; Totenhagen et al., 2018). Financial strain is associated with more negative and less positive interactions between partners, lower relationship satisfaction, and stability (Falconier and Epstein, 2010; Dew and Stewart, 2012; Falconier and Jackson, 2020), poorer communication between partners (Williamson et al., 2013), and diminished psychological well-being and increased depressive symptoms in the individual partners (Vinokur et al., 1996). Such findings indicate that parents of young children, who already face various challenges unique to this phase of life, may be particularly vulnerable when they experience stress from financial strain.

However, not all relationships may be affected equally by financial strain, as its impact may depend on the couple’s ability to mutually cope with stress. Financial strain in a family context, almost by definition, is a stressor that affects both partners. As such, it is a dyadic stressor. Hence, partners must often cope with both their own and their partner’s stress, which is known as dyadic coping (Bodenmann, 2005; Falconier and Kuhn, 2019). Dyadic coping refers to the processes in which both partners as a couple provide support for each other’s coping efforts, and/or both partners engage together in shared coping strategies to manage the stressor (Bodenmann et al., 2019). Couples can develop strategies together to deal with the situation, thereby reducing each other’s stress and improving mutual well-being as well as marital satisfaction (Bodenmann, 2005). Indeed, previous studies have shown that couples who engage in dyadic coping report higher marital satisfaction and marital stability (e.g., Bodenmann and Cina, 2006; Papp and Witt, 2010; Van Schoors et al., 2014, 2019; Karademas and Roussi, 2016; Falconier and Jackson, 2020).

Dyadic coping is a multifaceted construct that includes different types of coping. Specifically, Bodenmann et al. (2016, 2019) distinguished three forms of dyadic coping: positive coping, negative coping, and common dyadic coping. Positive dyadic coping refers to the partners’ perception that they assist each other by providing problem- and/or emotion-focused support and is the process by which partners take over responsibilities to reduce each other’s stress. Negative dyadic coping includes distancing oneself from one’s partner, blaming the partner for the stress, and providing support without empathy. Common dyadic coping refers to the process in which partners work together to handle stressful situations. Dyadic coping is usually measured with a self-report scale (i.e., Dyadic Coping Inventory, DCI; Bodenmann, 2008), assessing the various types of coping, as well as assessing the perception of partners that they can effectively communicate about the stress with each other, which is another essential aspect of dyadic coping. In the current research, we focus on the total score of the different subscales or facets of dyadic coping (i.e., general dyadic coping). These different facets of dyadic coping in combination refer to the partners’ sense that they are coping with challenges and stress together, or in other terms, that they are coping *dyadically*.

The stress-buffering effects of dyadic coping have been studied in light of a variety of stressors (e.g., work-related stress, illness; e.g., Falconier and Kuhn, 2019 for an overview), and some previous research has examined the association between financial stressors, dyadic coping, and the well-being of partners and their relationship. A study among Greek couples (Karademas and Roussi, 2016) found that financial strain undermined dyadic coping, which, in turn, decreased relationship satisfaction in both partners and increased distress but only among male partners. The authors did not report whether they tested a moderation model in which dyadic coping would reduce the negative impact of financial stress on relationship satisfaction and partners’ distress. A study by Falconier (2014) similarly found that a couples program to help couples cope with and manage financial stress promoted dyadic coping and increased the male partner’s relationship satisfaction. Another study, among low-income minority groups (Mitchell et al., 2015), showed that financial stress negatively affected relationship functioning, though when partners participated in a relationship education program, their dyadic coping increased; this, in turn, was associated with better relationship quality. Although these previous findings do not specifically focus on parents with children (i.e., the focus of the present research), they do show the impact financial strain can have on relationships, as well as the potential role of dyadic coping in mitigating this impact.

The goal of the present study was twofold. First, we sought to provide further support for the prediction that dyadic coping can buffer the impact of financial strain on the well-being of the relationship, as well as on the well-being of both partners. We examined this question in the context of young parents in West-Java. Studying financial stress and dyadic coping in parents of young children is important because the functioning of the relationship and the parents individually is strongly linked to the children’s well-being and development (e.g., Richter et al., 2018).

Second, we seek to contribute to the cross-cultural generalizability of previous findings in the field of dyadic coping. Most studies in this area have been conducted in Western countries (USA and Europe; see Falconier and Kuhn, 2019). A large-scale cross-cultural study in 35 countries found that the basic association between dyadic coping and relationship satisfaction varies between nations and that, in some countries, the association is higher for men than it is for women (Hilpert et al., 2016). In the current research, we examine our research questions among parents with young children in West Java, Indonesia. There, as compared to most Western countries, views on heterosexual marriages and parenting tend to be more traditional, with the husband holding primary responsibility for the family’s financial situation and the wife being more strongly involved in and responsible for child raising and the household (Zevalkink and Riksen-Walraven, 2001; Utomo, 2012; Riany et al., 2016). Moreover, West Java is an interesting area in which to study our research questions. This is because financial strain is a salient topic in many families, as indicated, for example, by the high divorce rate due to economic issues (Statistics of Jawa Barat, 2021; Izzah et al., 2022).

**Figure 1:**
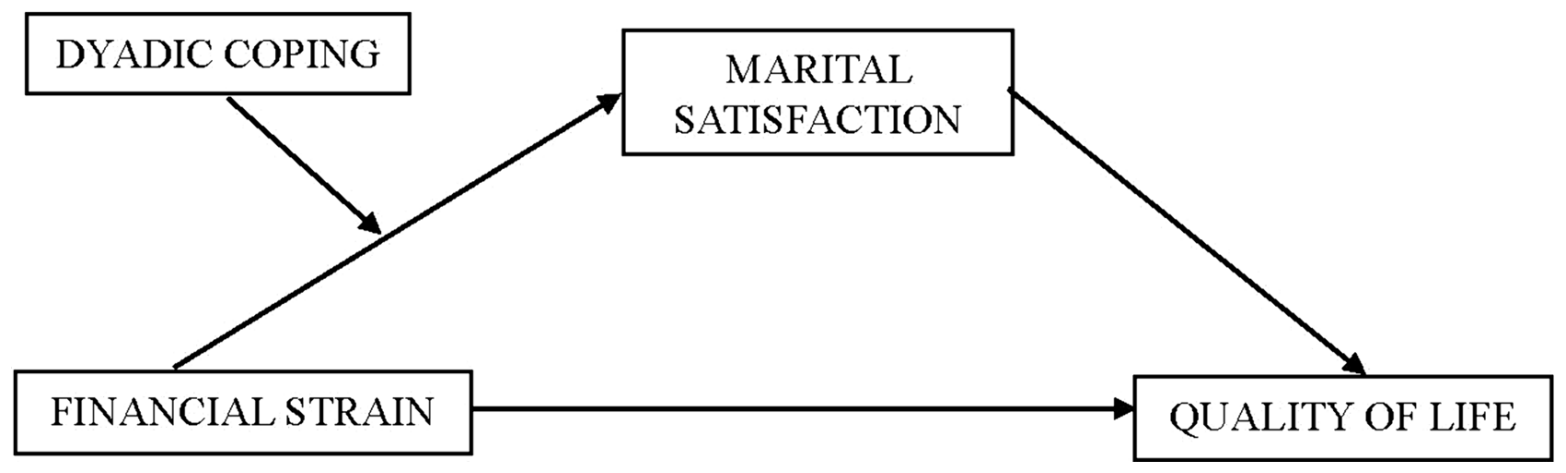
The model of variables.

Our research questions can be summarized as follows: (1) Can dyadic coping buffer the negative impact of financial strain on marital satisfaction, and (2) Can financial strain have an impact on quality of life via marital satisfaction? The research questions and predicted moderated mediated model are illustrated in Figure 1. We hypothesize that financial strain negatively affects marital satisfaction, and we hypothesize that dyadic coping moderates this association. That is, at higher levels of dyadic coping, the predicted negative association between financial strain and relationship satisfaction will be weaker. This hypothesis is based on previous findings on the role of dyadic coping in dealing with financial stress and stress more generally, as discussed above. As noted, financial strain is a ‘dyadic’ stressor, as it affects the couple and not just the individual partners, and thus requires *dyadic* coping. Moreover, we examine whether financial strain will negatively affect the well-being of the individual parents (as indicated by their reported quality of life) via relationship satisfaction. Previous research has shown that relationship satisfaction is a strong predictor of individual well-being (e.g., Whitton and Whisman, 2010; Gustavson et al., 2016). Given the previous findings that financial strain can have a negative impact on relationship quality, and given the general finding that relationship satisfaction is a strong predictor of individual well-being of partners, it stands to reason that the negative impact of financial strain on marital satisfaction in turn will be associated with lower individual well-being of both partners.

To test our predictions, we asked parents in West Java whose eldest children were aged 7–12 years to complete measures of financial strain, marital satisfaction, dyadic coping, and quality of life. Finally, we also explored gender differences. Although financial strain is a stressor that affects the couple, it is possible that the impact of financial strain is different for husbands and wives. For example, in West Java, men are generally the main financial provider in the family, and therefore the negative impact on individual well-being may be stronger, perhaps because men worry more about financial strain (*cf.* Karademas and Roussi, 2016).

## 2 Methods

The Ethics Committee of Universitas Padjadjaran approved the study (504/UN6.KEP/EC/2022). The study was pre-registered and can be accessed at https://osf.io/gh8nj/. All respondents signed the informed consent before completing the questionnaires.

### 2.1 Population and sample

This study focused on heterosexual married couples with school-aged children residing in West Java, Indonesia. We implemented two-stage cluster sampling. Five regions were randomly selected from 27 cities/districts in West Java (Kodinariya and Makwana, 2013) as the first stage cluster. The randomly selected areas were Bandung City, Garut Regency, Sukabumi City, Cirebon City, and Karawang Regency. Afterward, elementary schools were established as the second stage of the cluster through the involvement of public and private elementary schools in the selected areas. In total, 751 couples completed the questionnaire in full out of the 898 couples from 74 schools who participated in the study. We distributed the questionnaires at school, the participant read the instructions (or listened to the researcher’s explanation, e.g., https://youtu.be/dCLX46kdJK0), signed the inform consent, completed the questionnaire, and returned it in a sealed envelope to the teacher at their eldest child’s school.

### 2.2 Instruments

This study measured financial strain, dyadic coping, marital satisfaction, and quality of life and used paper and pencil self-report questionnaires. In this study, we used validated measures, namely, the WHOQOL-BREF (Purba et al., 2018a); Dyadic Coping Inventory (Rumondor et al., 2022); and Kansas Marital Satisfaction Scale—KMSS (Sorokowski et al., 2017). For financial strain measurement, an adaptation study was conducted for the Psychological Inventory of Financial Scarcity—PIFS (Van Dijk et al., 2021) following the guidelines of the ITC 2nd edition (International Test Commission, 2017).

#### 2.2.1 The Psychological Inventory of Financial Scarcity (PIFS)

PIFS is a self-assessment scale that measures the experience of financial scarcity through self-assessment of subjective perceptions of one’s financial situation, as well as affective and cognitive responses to these assessments. It consists of 12 items with a 7-point scale that measures (a) appraisals of insufficient financial resources (three items), (b) lack of control over one’s financial situation (three items), (c) response in the form of worrying or thinking about financial conditions continuously (three items), and (d) a short-term focus on one’s finances (three items). Together, these different facets assess participants’ overall level of financial stress, measured as a single construct (Van Dijk et al., 2021). Several items were formulated into statements, and respondents were asked to indicate the extent to which they agreed with the statement on a 7-point scale, with the far ends of the scale labeled 1 = strongly disagree (left) and 7 = strongly agree (right). The total score was used; the higher the total score, the more financial stress was experienced. The reliability of this measure was *α* = 0.91. Examples of the items were: “I often do not have enough money” (shortage of money) and “I often worry thinking about money” (rumination and worry).

#### 2.2.2 Dyadic Coping Inventory (DCI)

The DCI consisted of 37 items on a Likert scale of 1 = never (*very rarely*) to 5 = very often; this is a valid measuring instrument used among Indonesian respondents (Rumondor et al., 2022). The reliability value was *α* = 0.93. As noted in the introduction, dyadic coping is a multi-faceted construct consisting of the various related ways in which dyadic coping can be experienced in the relationship. The DCI consists of multiple subscales: stress communication (e.g., “I tell my partner openly how I feel and that I would appreciate his/her support”), positive dyadic coping (e.g., “I show empathy and understanding to my partner”), negative dyadic coping (e.g., “When my partner is stressed I tend to withdraw”), and common dyadic coping (e.g., “We help one another to put the problem in perspective and see it in a new lighting”) (Rumondor et al., 2022). A total dyadic coping score was computed following the instruction from Bodenmann (2008). Specifically, the total DCI score was obtained by adding up the scores for items number 1 to 35 after reversing the negative scores on items of negative dyadic coping (Bodenmann, 2008), items number 36 and 37 are evaluation items that are not included in the calculation of the total score. The DCI has a cut-off score: DCI is considered below average if the total DCI score is below 111, average if the total DCI score is 111–145; and a total score higher than 145 means that dyadic coping is above average (Bodenmann, 2008).

#### 2.2.3 Kansas Marital Satisfaction Scale (KMSS)

The KMSS (Schumm et al., 1983) consisted of 3 items that asked about the participants’ satisfaction with their wife/husband as a partner; satisfaction with their marriage; and satisfaction with the relationship with their partner. We also included an item about satisfaction with the partner’s role as a parent, i.e., “How satisfied are you with your partner as a parent?” (Chung, 2004). Participants responded to these items on a Likert scale ranging from 1 (very dissatisfied) to 7 (very satisfied). KMSS has proven to be applicable in many cultures; its reliability in the Indonesian context was *α* = 0.95 (Sorokowski et al., 2017). The total score was used as indicator of the level of marital satisfaction, by averaging all items (Schumm et al., 1983; Sorokowski et al., 2017). In this sample, *α* = 0.95.

#### 2.2.4 WHOQOL-BREF

To measure quality of life, we administered the WHOQOL-BREF (i.e., World Health Organization Quality of Life questionnaire, brief version), which was developed as a shortened version of the WHOQOL-100 (Skevington et al., 2004). The WHOQOL-BREF consisted of four domains, namely, physical (7 items), psychological (6 items), social (3 items), and environmental (8 items), plus 2 items regarding the overall quality of life and general health (The WHOQOL Group, 1998). Response options were Likert scales ranging from 1 (very bad/very unsatisfactory/not at all/never) to 5 (very good/very satisfactory/in excessive amounts/completely experienced/always). Examples of the items were “How satisfied are you with your ability to perform the activities of your daily life?” (physical domain), “How much do you enjoy life?” (psychological domain), “How satisfied are you with your personal relationships?” (social domain), and “How healthy is your physical environment?” (environmental domain).

To calculate the total score and mean of each domain, we converted the raw scores using the formula so that they were equivalent to the WHOQOL-100 score (Purba et al., 2018b). In the present study, the WHOQOL-BREF is reliable, with *α* = 0.71 (physical domain), *α* = 0.73 (psychological domain), *α* = 0.69 (social domain), and *α* = 0.84 (environmental domain).

#### 2.2.5 Personal data (demographic data)

Participants completed a series of personal questions concerning (1) age, (2) length of marriage, (3) education, (4) job, (5) number of children, (6) family income, expenditure, debt, and saving (7) religiosity (see the Sorokowski et al., 2017) and religious affiliation, (8) perceived level of country collectivism–individualism (see the Sorokowski et al., 2017). Religiosity was measure a single item (“Are you religious?”) and responses ranged from 1 (not at all) to 7 (extremely religious). Sample question of perceived level of collectivism–individualism was “In this society, aging parents generally live at home with their children.” Participants answered the question on a 7-point scale (from 1—strongly agree to 7—strongly disagree), a smaller number indicated a higher collectivism. We present the descriptives of these data in Tables 1, 2.

**Table 1:** Descriptive statistics (education, job, income, expenses, religion, debt, savings).

Demographic	Husband		Wife	
	Frequency	Percent	Frequency	Percent
Education				
Elementary (SD)	61	8.1	40	5.3
Junior high school (SMP)	91	12.1	117	15.6
Senior high school (SMA/SMK)	387	51.5	376	50.1
Diploma (D1/D2/D3)	64	8.5	65	8.7
Undergraduate	137	18.2	142	18.9
Postgraduate (S2/S3)	11	1.5	11	1.5
Job				
Permanent employed	316	42.1	129	17.2
Irregular employed	236	31.4	59	7.9
Entrepreneur	190	25.3	88	11.7
Retired	1	0.1	2	0.3
Student			1	0.1
Unemployed	8	1.1	471	62.7
Other			1	0.1
Family’s income (in million IDR)				
< 1	48	6.4	54	7.2
1.1–2	150	20.0	150	20.0
2.1–5	307	40.9	303	40.3
5.1–10	179	23.8	173	23.0
> 10	67	8.9	71	9.5
Family’s expenses (in million IDR)				
< 1	50	6.7	50	6.7
1.1–2	162	21.6	170	22.6
2.1–5	362	48.2	365	48.6
5.1–10	146	19.4	141	18.8
> 10	31	4.1	25	3.3
Religion				
Islam	705	93.9	706	94
Catholic	9	1.2	13	1.7
Protestant	33	4.4	31	4.1
Hindu	3	0.4	1	0.1
Penghayat Kepercayaan (native religions)	1	0.1		
Has savings/assets				
No	223	29.7	220	29.3
Yes	528	70.3	531	70.7
Has debts				
No	227	30.2	251	33.4
Yes	524	69.8	500	66.6

**Table 2:** Descriptive statistics (age, length of marriage, number of children).

Demographic	*N*	Minimum	Maximum	Mean	Std. Deviation
Age (husband)	751	23	59	37.53	5.09
Age (wife)	751	22	59	34.42	4.85
Length of marriage (husband)	751	6	22	10.98	2.21
Length of marriage (wife)	751	7	22	10.99	2.20
Number of children (husband)	751	1	5	1.97	0.70
Number of children (wife)	751	1	5	1.97	0.70
Religiosity (husband)	751	1	7	5.20	1.37
Religiosity (wife)	751	1	7	5.26	1.31
Culture (husband)	751	10	43	17.91	6.02
Culture (wife)	751	10	65	18.30	6.50

### 2.3 Data analysis

**Figure 2:**
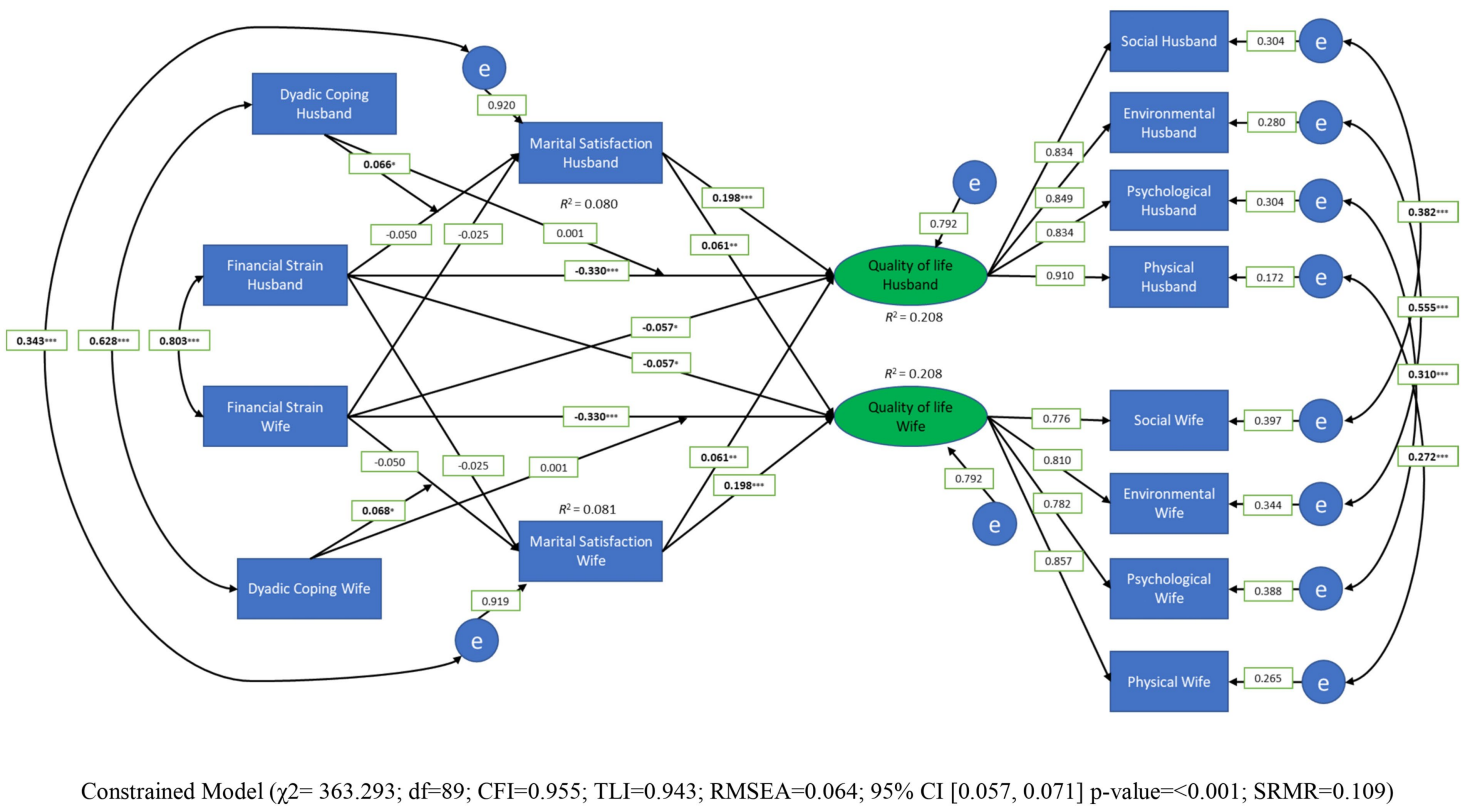
Moderated mediated model of dyadic coping, financial strain, marital satisfaction, and quality of life (constrained model without control variable).

The study examined the moderating effect of dyadic coping on the relationship between financial strain and marital satisfaction for both husband and wife; and the mediating effect of marital satisfaction on the relationship between financial strain and quality of life for both husband and wife, as well as the actor-partner effect of financial strain on quality of life. We used the actor-partner interdependence model (APIM; Kashy and Kenny, 1999) analysis to investigate the relationship between the variables and test our predictions. The APIM is a form of a structural equation modeling that allows for the measurement of actor and partner effects. “Actor effects” refers to the impact of an individual’s characteristics on their own outcomes, while “partner effects” refers to the impact of their partner’s characteristics on their outcomes (Cook and Kenny, 2005). Structural equation modeling (SEM) was conducted to test the dynamic model of the relationship between financial strain, quality of life, marital satisfaction as a mediator and dyadic coping as a moderator variable (see Figure 2); it was conducted using the lavaan, psych, and semplot packages in *R. Maximum* likelihood was used as the estimator for SEM analysis, and the model fit indices were evaluated based on the criteria from Hu and Bentler (1999), see also Kenny (2020). We explored gender differences through comparing the saturated model, where paths were allowed to vary freely, with a model where actor and partner effects were constrained to be equal across gender (see Ledermann et al., 2011; Xu et al., 2016); using a chi-squared test for differences, the constrained model did not worsen or improve the model fit compared to the unconstrained model, suggesting that the model was not different for men and women. Thus, we report the constrained model.

**Figure 3:**
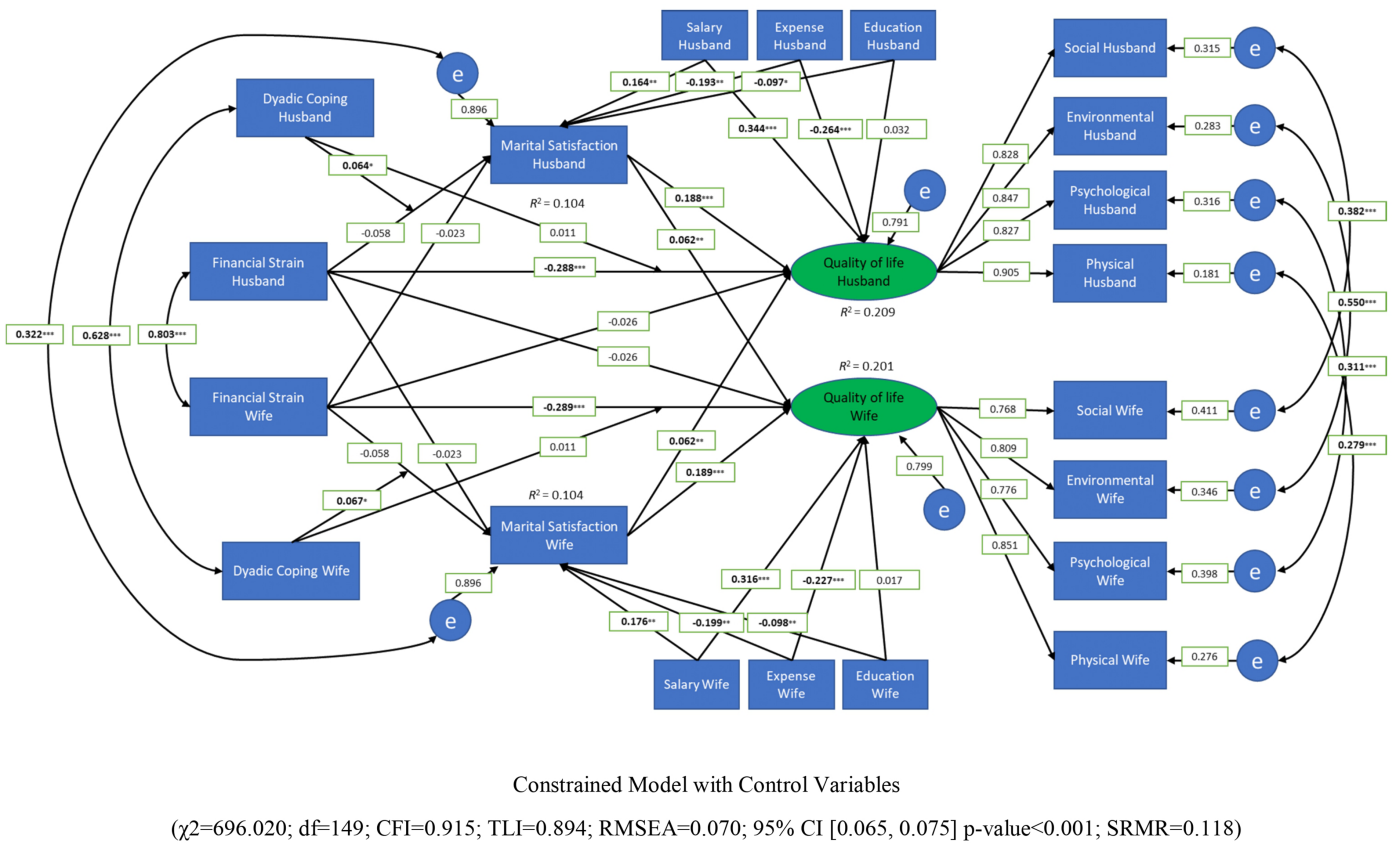
Moderated mediated model of dyadic coping, financial strain, marital satisfaction, and quality of life (constrained model with control variable).

We also explored the model using three control variables (e.g., family income, expenses, education; see Figure 3). In the Figures 2, 3 the measured (observable) variables are total dyadic coping, marital satisfaction, financial strain, and four domains of quality of life; quality of life was used as a latent variable. We used the factor score in SEM to minimize the error of the measurement and gather a better estimation model (see McNeish and Wolf, 2020).

## 3 Results

### 3.1 Descriptive statistics

In total, data from 751 couples were used in the analysis. Demographics and other descriptive information can be seen in Tables 1, 2. The majority of the respondents were in their middle adulthood years (mean age husband = 37.5 years; SD = 5.09 and wife = 34.4 years; SD = 4.85), and the mean length of marriage was 11 years; the average number of children was 2; the mean age of the eldest child was 9.5 years (SD = 1.76). Participants had generally completed high school; husbands were generally employed; wives were generally not employed; income and expenses were generally between 2.1–5 million Indonesian Rupiah. Participants’ income was in line with the typical income of Indonesians (BPS-Statistics Indonesia, 2022), and, based on expenditures, participants were generally middle class (Suhada and Swasono, 2023; Purwanto, 2024). Most were Muslim. They mostly had savings but also debts. Most of the participants believed that they lived in a collective culture and were quite religious.

### 3.2 Mean, standard deviation, and interrelations of variables

The mean of financial strain was generally moderate (*M* = 38.92 *SD* = 19.07; *M* = 37.8 *SD* = 18.22); the husband’s financial strain was significantly higher than the wife’s (*t* (750) = 2.63; *p* = 0.01). Compare to the cut-off score (Bodenmann, 2008), the mean of dyadic coping was generally below average (*M* = 94.73 *SD* = 14.83; *M* = 95.56 *SD* = 14.91); but the wife’s dyadic coping was significantly higher than the husband’s (*t* (750) = 2.42; *p* = 0.02). The mean of marital satisfaction was generally high (*M* = 26.41 *SD* = 2.93; *M* = 25.35 *SD* = 4.63); the husband’s marital satisfaction was significantly higher than the wife’s satisfaction (*t* (750) = 6.55; *p* = 0.00). Husbands’ and wives’ quality of life was similar in all four domains (mean of physical domain 69.60 and 68.75; psychological 71.02 and 69.77; environment 62.03 and 62.62; social 68.65 and 67.44).

Table 3 shows all correlations between variables. For both husbands and wives, there was a significant negative correlation between financial strain and marital satisfaction (*r* = − 0.13; *r* = −0.12), as well as between financial strain and dyadic coping (*r* = −0.19; *r* = −0.21). There was a significant positive correlation between marital satisfaction and dyadic coping for both husbands and wives (*r =* 0.27; *r =* 0.25) and between marital satisfaction and the four quality of life domains (*r =* 0.24, 0.26, 0.21, 0.24; *r =* 0.31, 0.31, 0.35, 0.36). There was a significant association between income and other variables (see Table 3). As would be expected, the husband’s and wife’s income were strongly correlated, *r* = 0.94. Moreover, both the husband’s as well as wife’s level of financial strain were negatively associated with their income, both *r’*s = −0.43. Income had a weak positive correlation with both the husband’s and wife’s dyadic coping, *r’*s = 0.18 and 0.23, respectively.

**Table 3:** Interrelations of financial strain, dyadic coping, marital satisfaction, and quality of life.

		1	2	3	4	5	6	7	8	9	10	11	12	13	14	15	16
1	Income (husband)	1															
2	Financial strain (husband)	−0.43^**^	1														
3	Dyadic coping (husband)	0.18^**^	−0.19^**^	1													
4	Marital satisfaction (husband)	0.02	−0.13^**^	0.27^**^	1												
5	Quality of life - physical (husband)	0.19^**^	−0.31^**^	0.42^**^	0.24^**^	1											
6	Quality of life - psychological (husband)	0.19^**^	−0.32^**^	0.42^**^	0.26^**^	0.70^**^	1										
7	Quality of life - environmental (husband)	0.40^**^	−0.47^**^	0.42^**^	0.21^**^	0.73^**^	0.70^**^	1									
8	Quality of life - social (husband)	0.18^**^	−0.28^**^	0.41^**^	0.24^**^	0.74^**^	0.61^**^	0.67^**^	1								
9	Income (wife)	0.94^**^	−0.41^**^	0.19^**^	0.01	0.18^**^	0.18^**^	0.38^**^	0.17^**^	1							
10	Financial strain (wife)	−0.43^**^	0.81^**^	−0.15^**^	−0.06	−0.25^**^	−0.26^**^	−0.42^**^	−0.23^**^	−0.44^**^	1						
11	Dyadic coping (wife)	0.21^**^	−0.20^**^	0.75^**^	0.17^**^	0.31^**^	0.33^**^	0.34^**^	0.32^**^	0.23^**^	−0.21^**^	1					
12	Marital satisfaction (wife)	0.05	−0.15^**^	0.18^**^	0.38^**^	0.16^**^	0.20^**^	0.18^**^	0.17^**^	0.06	−0.12^**^	0.25^**^	1				
13	Quality of life - Physical (wife)	0.16^**^	−0.26^**^	0.26^**^	0.09^*^	0.56^**^	0.48^**^	0.50^**^	0.45^**^	0.17^**^	−0.28^**^	0.31^**^	0.17^**^	1			
14	Quality of life - psychological (wife)	0.14^**^	−0.25^**^	0.28^**^	0.16^**^	0.44^**^	0.57^**^	0.46^**^	0.38^**^	0.15^**^	−0.32^**^	0.31^**^	0.26^**^	0.66^**^	1		
15	Quality of life - environmental (wife)	0.37^**^	−0.44^**^	0.29^**^	0.08^*^	0.51^**^	0.49^**^	0.70^**^	0.45^**^	0.38^**^	−0.47^**^	0.35^**^	0.24^**^	0.68^**^	0.63^**^	1	
16	Quality of life - social (wife)	0.19^**^	−0.26^**^	0.30^**^	0.16^**^	0.47^**^	0.41^**^	0.47^**^	0.57^**^	0.19^**^	−0.28^**^	0.36^**^	0.24^**^	0.68^**^	0.54^**^	0.64^**^	1
	Mean	–	38.92	94.73	26.41	69.60	71.02	62.03	68.65	–	37.80	95.56	25.35	68.75	69.77	62.62	67.44
	SD	–	19.07	14.83	2.93	13.96	14.17	14.17	14.45	–	18.22	14.91	4.63	13.15	2.53	12.73	13.61

*p < 0.05; **p < 0.01.

In general, the relationship between variables in husbands and wives was positive and strong; an example is the correlation between the financial strain of husbands and wives (*r =* 0.81) and the dyadic coping of husbands and wives (*r =* 0.75). Moderate correlations were found in the relationship between husbands’ and wives’ marital satisfaction (*r =* 0.38). In the four domains of quality of life, there were moderate correlations between husbands and wives (*r =* 0.56 for physical, 0.57 for psychological, 0.70 for environment, 0.57 for social).

### 3.3 The moderating effect of the total score of dyadic coping and the mediating effect of marital satisfaction

The chi-square difference test shows that the model with constraining the paths to be equal across gender did not decrease model fit than the model allowing all paths to be freely estimated, *χ^2^* (8) = 6.187, *p* = 0.63. Thus, the constrained model fitted the data well (*χ^2^* = 363.293, *df* = 89, CFI = 0.96, TLI = 0.94, RMSEA = 0.06, CI [0.06, 0.07], *p* < 0.001; and *SRMR* = 0.11), suggesting the effects were similar for husband and wives. The model is depicted in Figure 2. The model showed that husbands’ and wives’ financial strain had no direct effect on their own marital satisfaction—actor effects (*γ* = −0.050, 95% CI [−0.118, 0.020], *p* = 0.160) and their partner marital satisfaction—partner effects (*γ* = −0.025, 95% CI [−0.093, 0.045], *p* = 0.487). However, addressing our main research question, total dyadic coping moderated the association between financial strain and marital satisfaction, for both husbands and wives, (*β* = 0.066; 95% CI [0.014, 0.116]; *p* = 0.01; *β* = 0.068; 95% [0.014, 0.116]; *p* = 0.01, respectively). Moreover, for husbands and wives, there were no mediation effects of marital satisfaction on association of their quality of life and financial strain, both for actor effects (*β* = −0.01; 95% CI [−0.027, 0.005]; *p* = 0.166) and partner effects (*β* = −0.001; 95% CI [−0.008, 0.004]; *p* = 0.502). See Table 4 for the details. However, financial strain experienced by husbands and wives had a negative direct impact on their own quality of life (*β* = −0.330, 95% CI [−0.439, −0.305]; *p* < 0.001) and affected the quality of life of their partners (*β* = −0.057, 95% CI [−0.127, −0.001]; *p* < 0.047). Finally, both husbands’ and wives’ marital satisfaction had a positive effect on their own quality of life (*β* = 0.198, 95% CI [0.171, 0.277]; *p* < 0.001) and the quality of life of their partner (*β* = 0.061, 95% CI [0.018, 0.120]; *p* < 0.008).

**Table 4:** Direct effect and indirect effect for the actor partner mediation moderation model.

Effects	Estimate	SE	*z*	*p*	95 CI
Direct effects					
Actor effects					
FS – QoL	−0.330^***^	0.034	−11.088	< 0.001	[−0.439, −0.305]
MS – QoL	0.198^***^	0.027	8.395	< 0.001	[0.171, 0.277]
FS – MS	−0.050	0.035	−1.405	0.160	[−0.118, 0.020]
Partner effects					
FS – QoL	−0.057^*^	0.032	−1.990	0.047	[−0.127, −0.001]
MS – QoL	0.061^**^	0.026	2.637	0.008	[0.018, 0.120]
FS – MS	−0.025	0.035	−0.695	0.487	[−0.093, 0.045]
Indirect effects					
Actor effects					
FS – MS – QoL	−0.010	0.008	−1.386	0.166	[−0.027, 0.005]
Partner effects					
FS – MS – QoL	−0.001	0.003	−0.672	0.502	[−0.008, 0.004]
Moderation effect					
DC * MS (Husband)	0.066^*^	0.026	2.561	0.010	[0.014, 0.116]
DC * MS (Wife)	0.068^*^	0.026	2.561	0.010	[0.014, 0.116]
DC * QoL	0.001	0.027	0.036	0.971	[−0.052, 0.054]

^*^p < 0.05; ^**^p < 0.01; ^***^p < 0.001. FS, Financial Strain; MS, Marital Satisfaction; DC, Dyadic Coping; QoL, Quality of Life.

**Figure 4:**
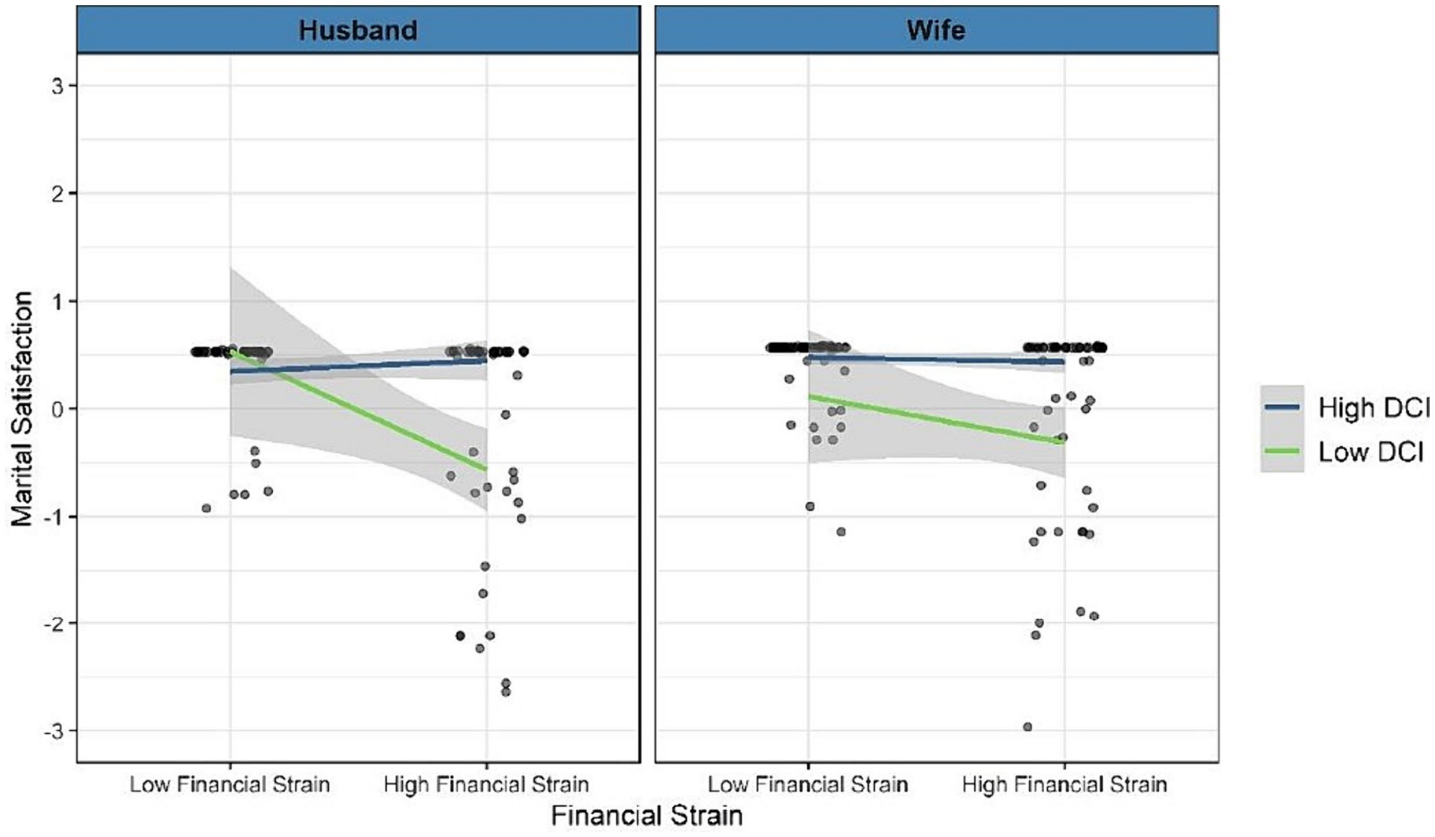
Moderation effect of total dyadic coping on the association between financial strain and marital satisfaction.

Figure 4 shows the significant moderation effects of dyadic coping. For husbands, at low levels of dyadic coping, the association between financial strain and marital satisfaction was negative (albeit marginally; *B* = −0.946, *p* = 0.093). At high levels of dyadic coping, this association was weaker and not significant (*B =* 0.115, *p* = 0.367). For wives, at low levels of dyadic coping, the association between financial strain and marital satisfaction was negative (albeit non-significant: *B* = −0.390, *p* = 0.397); at high levels of dyadic coping the slope was weaker (*B* = −0.04, *p* = 0.509). The significant moderation indicates that the slopes for high versus low dyadic coping differ significantly. As can be seen in Figure 4, although the simple slopes were non-significant, the pattern is consistent with the buffering-by-dyadic coping hypothesis: the link between financial strain and marital satisfaction was weaker at higher levels of dyadic coping than at lower levels of dyadic.

To further explore the data, we tested the model controlling for variables (i.e., family income and expenses, educational background) that were significantly associated with the measured variables (e.g., marital satisfaction and quality of life). The results can be found in the Figure 3. Adding these variables did not change the conclusions of the role of financial strain and dyadic coping, the main findings were very similar. Furthermore, we also ran the models using each subscale of dyadic coping. For the sake of brevity, and because we did not have *a priori* predictions about this, we do not report the findings here. However, the first author can be contacted for more details about these models, and the data will be publicly available at the OSF.^1^

## 4 Discussion

The present study examined whether, among parents with young children in West Java, Indonesia, dyadic coping can buffer the negative impact of financial strain on the marital satisfaction and individual well-being of both parents. Consistent with this main prediction, the association between the husband’s experienced financial strain and his marital satisfaction was moderated by his experienced dyadic coping. However, we did not find that financial strain directly affected marital satisfaction, although the pattern of the moderation effect suggested that lower levels of dyadic coping strengthen the link between financial strain and marital satisfaction. Moreover, financial strain was negatively associated with the quality of life of both wives and husbands. We did not find that marital satisfaction mediated the link between financial strain lower quality of life. In short, extending previous research done mostly in Western countries, the present research among Javanese couples shows that financial strain negatively impacts particularly the individual well-being of both partners, and we found only weak support that marital satisfaction is more strongly affected by financial strain to the extent that dyadic coping is lower.

The predicted moderation effects occurred for both husbands and wives. Comparing the unconstrained and constrained models indicated that the effects were not significantly different for husband and wives, however, the pattern seemed somewhat stronger for husbands (notably, in the unconstrained model, the moderation was significant for husbands but not for wives). This is in line with some previous work showing that the effects of dyadic coping on dealing with financial stress is particularly strong among males (Falconier, 2014; Karademas and Roussi, 2016). One explanation is that men may feel more responsible for finances in the family, either because they provide the largest part of the income (indeed, most husbands in this study were employed, while most wives were not) and/or because they experience societal norms in which the husband is the main provider for the family. In fact, in Indonesia, as established in the Law of the Republic of Indonesia on Marriage (1974), a husband holds primary responsibility for providing for his wife and children. At the same time, as a study by Fernandez et al. (2015) showed, married couples in Indonesia tend to make joint financial decisions concerning issues like savings, household expenditures, gift-giving, and child-related financial matters such as food, school, and health. A previous study in Indonesia found that husbands’ subjective well-being is higher when they are not solely responsible for financial and other types of decisions (Fernandez et al., 2015). Thus, while we should interpret the seemingly stronger pattern for husbands than wives with caution given that the models for husbands and wives were not significantly different, given the consistency with previous findings, it seems important to consider the potentially more important role of dyadic coping for husbands as compared to wives when dealing with financial stress. Future research should further examine such possible gender differences.

We found actor effects between financial strain and quality of life for both men and women. This finding is in line with other studies on the negative impact of economic strain on the quality of life of family members (e.g., Pahlevan Sharif et al., 2021; Fonseca et al., 2023). We also found partner effects such that both the one partner’s experienced financial strain negatively affected the partner’s quality of life. The findings are consistent with the idea that one might expect that such processes occur between partners because of the dynamics within the marriage. The fact that the partner effects were much weaker than the actor effects is consistent with previous meta-analyses showing that, in dyadic research, actor effects appear to be much more common and easy to find than partner effects (Joel et al., 2020). As with the lack of direct actor effects, we did not find partner effects for the relationship between financial strain and marital satisfaction.

### 4.1 Strengths and limitations

We highlight several strengths of the present research. The current research was conducted in West Java using a large sample. This is an important contribution because it shows that previous findings can be (at least partially) generalized to a country with a different cultural background and different views on marriage, in which partners tend to have different roles (e.g., Falconier and Kuhn, 2019; Kelley et al., 2023; Totenhagen et al., 2023). Moreover, we conducted our research among parents whose eldest child was aged 7–12 years old and found that financial strain is negatively associated with both parents’ quality of life. This is an important finding because the parents’ quality of life may impact the development and well-being of children (e.g., Jozefiak et al., 2008; Shek, 2008, 2021; Conger et al., 2010; Heintz-Martin and Langmeyer, 2020; Liu et al., 2023; Riany and Morawska, 2023). Previous research has shown that financially stressed parents experience more conflict with their partners and use harsh parenting styles with their children, which affects their children’s well-being. The use of dyadic coping by parents in their marital relationship has a positive effect on the well-being of their children (Zemp et al., 2016).

As to the limitations, our sample was not representative of the West Javanese population, and it might also contain a selection bias (e.g., couples very low in marital satisfaction may be reluctant to participate in a study on marriage). Therefore, generalizing the results to the Indonesian population requires caution, as Indonesia consists of a large variety of cultures. Second, our findings are based on a cross-sectional study, and we therefore cannot make causal inferences. An additional limitation of the cross-sectional design is that the salience of one’s current financial state, when filling in the questionnaires, may have had an unrealistic impact on participants’ reports of their marital satisfaction and individual well-being, possibly leading to an overestimation of the effects. It is important for future research to examine similar research questions using longitudinal designs with more time points. Third, the level of financial strain that our participants reported was relatively moderate; thus, it is worth comparing the results of this study with those from a group of participants who experience particularly high levels of financial strain. For example, an interesting question is whether the buffering effect of dyadic coping on relationship satisfaction, as we found among males, may “break down” at very high levels of financial stress. Fourth, our sample consisted mostly of Muslims who were quite religious. Previous studies in Indonesia have shown that higher levels of religiosity are associated with a lower risk of high financial stress levels and with a higher quality of life for wives/mothers (Azzara et al., 2022). Religiosity may have been an important buffer against financial stress in our sample, which itself may explain why dyadic coping did not have the strong buffering effects one might expect. In general, religiousness has been an important complementary way of coping with stressful situations (e.g., Arshad and Bibi, 2024). An interesting avenue for future research is to test the combined influence of religiosity and dyadic coping on stress, marital satisfaction, and quality of life for both partners.

## 5 Conclusion

Indonesia is one of the largest countries in the world, with a relatively high prevalence of financial and marital stress. We found that for dyadic coping moderated the relationship between financial strain and marital satisfaction, although these effects were not strong and even at high dyadic coping the link between financial strain and marital satisfaction was relatively weak and non-significant. The impact of financial strain on both own and the partner’s individual wellbeing were stronger and clearer. As such, the present research contributes to our understanding how financial stress affects both partners, their marriage, and their individual well-being (and perhaps, ultimately, their children) in Javanese couples. These findings are interesting for researchers as well as practitioners. Different stakeholders, for example, government institutions (e.g., the office of religious affairs), religious organizations (e.g., church), and non-governmental organizations (e.g., marriage and family counselor), are advised to focus more on teaching effective coping strategies to couples facing financial stress and showing them how to practice dyadic coping. We hope that practitioners are inspired to think of ways to implement the present findings into prevention and intervention measures, and that other researchers (in Indonesia and across the globe) will further examine how financial stress can negatively affect parents and children and what factors could prevent this.

## Data Availability

The raw data supporting the conclusions of this article will be made available by the corresponding author upon reasonable request.

## Footnotes

^1^ https://osf.io/2af34
